# Brain-associated alterations of Hippo pathway transcription in early maturing Atlantic salmon

**DOI:** 10.1186/s12862-025-02398-4

**Published:** 2025-05-26

**Authors:** Ehsan Pashay Ahi, Jukka-Pekka Verta, Johanna Kurko, Annukka Ruokolainen, Pooja Singh, Paul Vincent Debes, Jaakko Erkinaro, Craig R. Primmer

**Affiliations:** 1 Organismal and Evolutionary Biology Research Programme, Faculty of Biological and Environmental Sciences, University of Helsinki, Viikinkaari 9, Helsinki, 00014 Finland; 2 Faculty of Biosciences and Aquaculture, Nord University, Bodø, Norway; 3 Department of Aquatic Ecology, Institute of Ecology and Evolution, University of Bern, Bern, 3012 Switzerland; 4 Center for Ecology, Evolution & Biogeochemistry, Swiss Federal Institute of Aquatic Science and Technology (EAWAG), Kastanienbaum, Switzerland; 5 Department of Aquaculture and Fish Biology, Hólar University, Hólar, Iceland; 6 Natural Resources Institute Finland (Luke), P.O.B. 412, Oulu, FI-90014 Finland; 7 Institute of Biotechnology, Helsinki Institute of Life Science (HiLIFE), University of Helsinki, Helsinki, Finland

**Keywords:** Gene expression, Atlantic salmon, *vgll3*, Hippo pathway, Brain, Early maturation

## Abstract

**Background:**

Pubertal timing is a key life history trait, shaped by ecological pressures to balance reproductive success and survival. Emerging evidence suggests a link between adiposity and early maturation, potentially through hormonal signaling pathways governing puberty timing. The timing of sexual maturation in Atlantic salmon has a strong genetic basis in addition to being linked with environmental shifts and lipid reserves. A gene encoding a co-factor of the Hippo pathway, *vgll3*, is a major determinant of maturation timing in salmon. The Hippo pathway is known for its evolutionary conserved molecular signal role in both sexual maturation and adipogenesis.

**Results:**

In this study, we tested the expression of Hippo pathway genes in the brain of immature and mature male Atlantic salmon carrying either the *early* or the *late* maturation genotype of *vgll3.* We found increased brain expression of a major Hippo pathway kinase (*lats1b*) in individuals with *early* maturation genotypes of *vgll3* before maturation development of testes was evident. Moreover, we found components and regulating partners of the Hippo pathway showing differential expression in brain of individuals with *early* and *late vgll3* genotypes prior to maturation. This may suggest a role for the Hippo pathway in central nervous system processes that regulate the preparation for maturation.

**Conclusions:**

This study characterizes transcriptional changes in components of the Hippo pathway in the brain in relation to *vgll3*-mediated early maturation in Atlantic salmon, highlighting the potential involvement of this pathway in the central regulation of maturation prior to gonadal development.

**Supplementary Information:**

The online version contains supplementary material available at 10.1186/s12862-025-02398-4.

## Background

Early maturation, or precocious puberty, involves the early onset of puberty marked by physical and hormonal changes. It is associated with physical and psychological challenges and an increased risk of metabolic and cardiovascular disorders later in life [1–3]. Evidence suggests that adiposity, characterized by excess body fat, may influence hormonal pathways regulating puberty timing [4], though the underlying mechanisms remain poorly understood [3]. Fish models have become invaluable tools for studying metabolic and sexual maturation processes, offering insights into ecological and evolutionary aspects of these phenomena. Their conserved endocrine systems and genetic similarities to mammals provide a foundation for investigating shared molecular pathways governing metabolic and reproductive regulation [5]. The onset of sexual maturation, initiated in the brain by the release of gonadotropin-releasing hormone (GnRH) from the hypothalamus, exemplifies an evolutionarily conserved mechanism triggering downstream hormone production by the pituitary gland; luteinizing hormone (LH) and follicle-stimulating hormone (FSH), and subsequent activation of gonads [6]. However, the regulation of pubertal timing in fish goes beyond the hypothalamus-pituitary-gonad (HPG) axis, incorporating interactions with other brain regions, such as the pineal gland, amygdala, and preoptic area, which respond to environmental stimuli [7]. Fish brains, reflecting their diverse evolutionary history and aquatic adaptations, are less centralized anatomically compared to mammalian brains, with specific regions playing unique roles in puberty onset [8, 9]. For instance, the preoptic area has a more prominent role in GnRH production in fish, while the pineal gland is pivotal in regulating reproductive behaviors and timing, particularly through photoperiodic responses in species like salmonids [10–13]. These adaptations highlight the ecological significance of reproductive timing, shaped by environmental conditions such as light cycles, to optimize reproductive success in variable aquatic ecosystems. A more holistic approach to studying fish brains, beyond the classical focus on the HPG axis, could uncover pre-maturation molecular signals in less-explored regions of the brain. These signals might trigger the HPG axis and initiate puberty in response to environmental cues. Identifying such early molecular markers not only deepens our understanding of sexual maturation from an evolutionary perspective but also has practical implications. In wildlife conservation, selective breeding, and studies of ecological resilience, these markers could help predict shifts in reproductive patterns or facilitate interventions to address challenges posed by changing environments.

The timing of sexual maturation in Atlantic salmon is strongly connected with seasonal shifts in the environment and body condition [14]. The allocation of lipids has a pivotal role influencing salmon age at maturity, as salmon require a specific lipid reserve threshold to initiate maturation [15]. Notably, within Atlantic salmon, a single locus encompassing the gene vestigial-like family member 3 (*vgll3*) stands as the principal genetic determinant of maturation timing, associated with over 39% of the variance in age at maturity in natural populations [16, 17]. The impact of *vgll3* on age at maturity is sex-specific and becomes evident in male salmon as early as one year old under controlled conditions [18–21]. Intriguingly, its human ortholog (*VGLL3*) has also demonstrated an association with age at maturity [22, 23]. In addition to its role in maturation age, Vgll3 serves as an inhibitor of adipogenesis in mice [24]. In salmon, individuals with distinct *vgll3* genotypes exhibit seasonal variations in energy storage, suggesting a link between energy utilization and environmental changes [14, 25, 26].

Recent investigations in Atlantic salmon have demonstrated a robust correlation between *vgll3* alleles associated with *early* (E) and *late* (L) maturation and the expression patterns of a number of crucial reproductive axis genes [27, 28]. The anticipation that *vgll3* genotype effects on reproductive axis genes may be modulated through divergent Hippo signaling pathway activation has been shown by gene co-expression network analysis [29]. The Hippo pathway is rapidly emerging not only as a pivotal molecular signal governing sexual maturation in vertebrates [30–32] but also as a regulator in the equilibrium between adipocyte proliferation and differentiation [25]. Moreover, the Hippo pathway seems to play an integral role in transducing environmental cues, such as alterations in dietary fat content and temperature, into transcriptional responses [33–36]. Whilst *vgll3* expression level has been negatively associated with adipocyte differentiation [24] and gonad development [20], within the Hippo pathway, VGLL3 operates as a significant transcription co-factor, predominantly assuming an activating role [37]. This dynamic interplay involves competition with another major transcription co-factor of the pathway, YAP1, which primarily functions as an inhibitor of the Hippo pathway [31, 37]. YAP1 is also involved in adipogenesis and neural development [25, 38, 39]. Collectively, these findings highlight that Atlantic salmon is a potential natural model for pubertal timing since its distinct *vgll3* alleles provide a functional connection between maturation and adipogenesis, and offer an opportunity to study the direct molecular mechanisms interlinking sexual maturation and energy acquisition. In this study, we sought to investigate how different *vgll3* genotypes influence transcriptional activity in the brain both before and after sexual maturation. The study examined males (since the maturation occurs on average at earlier age in salmon males than females) with different maturation phenotypes (mature and immature) and genotypes (*early* vs. *late* maturing *vgll3* genotypes).

## Materials and methods

### Fish material and tissue sampling

Individuals used in the study were from the same population (Oulujoki) and cohort used in a previous study [20]. At eight months of age, fish were individually tagged with passive integrated transponders (PIT-tags) and a fin clip was collected for genotyping. Genotyping was performed using a set of 141 SNP markers, including *vgll3*, and PCR-sequencing [40]. Individuals were assigned to families using SNPPIT [41]. This provided access to individually PIT tagged individuals with known *vgll3* genotypes (see Verta et al., (2020) for more details of crossing and rearing). For this study, male individuals were sampled at the ages of 1.5 to 2 years [42]. Fish were euthanized by an overdose of MS222 (400 mg/L) and the entire brain (including the pituitary gland) of individual males was sampled at one of three time points, each reflecting a different maturation development stage as described below:

*Immature 1*-stage individuals were collected during the late spring period (5–21 May), exhibiting an average mass of 17.5 ± 5.5 g and an average length of 12.1 ± 1.3 cm. These individuals displayed no discernible signs of gonad development, as indicated by a gonadosomatic index (GSI) value of 0. The GSI is a ratio of gonad weight to total body weight, commonly used as an indicator of reproductive development and maturity in fish.

*Immature 2*-stage individuals were sampled in the summer season (4–17 July), with an average mass of 33.7 ± 15.8 g and an average length of 17.3 ± 1.3 cm. Some of these individuals exhibited initial indications of the onset of phenotypic maturation processes, as reflected by GSI values ranging from 0.0 to 0.4.

*Mature*-stage individuals were collected during the anticipated spawning period in early autumn (1–15 October). These individuals had an average mass of 82.7 ± 20.2 g and an average length of 20.8 ± 1.6 cm. All individuals in this group displayed well-developed gonads indicative of maturity, with a GSI exceeding 3.

Subsequently, these tissue samples were rapidly frozen using liquid nitrogen and stored at a temperature of -80 °C until they were processed further.

### RNA extraction

RNA was extracted from a total of 24 samples of male brain (four homozygote individuals of *vgll3 early*; vgll3*EE, and four of *vgll3 late*; vgll3*LL) for each of the three time points) using a NucleoSpin RNA kit (Macherey-Nagel GmbH & Co. KG). The collected samples were transferred to tubes containing 1.4 mm ceramic beads from Omni International, along with 350 µl Buffer RA1. Homogenization was performed using the Bead Ruptor Elite (Omni International) at a frequency of 30 Hz for a total of 2 min (in 6 cycles of 20 s each). The subsequent RNA extraction procedures adhered to the guidelines provided by the manufacturer, with the kit also including an integrated DNase stage to eliminate any remaining genomic DNA (gDNA). At the conclusion of this process, the RNA extracted from each individual sample was eluted using 50 µl of nuclease-free water. To assess RNA quantity, measurements were taken using the NanoDrop ND-1000 instrument (Thermo Scientific, Wilmington, DE, USA), while the quality of the RNA was evaluated through the employment of the 2100 BioAnalyzer system (Agilent Technologies, Santa Clara, CA, USA). The RNA integrity number (RIN) exceeded 7 for all samples. For the hybridization step within the NanoString panel, a total of 100 ng of the extracted RNA from each isolation was utilized.

### NanoString nCounter mRNA expression panel

In this study, the NanoString panel of probes was an expanded iteration of the panel used in a previous study [31], which initially focused on investigating age-at-maturity-related gene expression in Atlantic salmon. This updated panel, encompassing more than 100 additional genes (333 target and 9 reference genes), particularly concentrates on an extensive collection of the Hippo pathway components and other genes directly interconnected with the Hippo pathway (supplementary file 1). The gene selection process involved both literature review and the utilization of tools [31], such as the IPA (Ingenuity Pathway Analysis) from Qiagen, as well as other accessible web-based tools and databases; KEGG and PANTHER [43–45]. Notably, the panel includes probes targeting genes linked to age-at-maturity in Atlantic salmon, such as *vgll3a* and *six6a* along with their corresponding paralogs *vgll3b* and *six6b*. Additionally, the panel incorporates probes for other genes functionally associated with sexual maturation, including those within the hypothalamus-pituitary-gonadal (HPG) axis. Given the presence of multiple paralogs for most candidate genes due to both the teleost-specific and salmonid-specific whole-genome duplication events, all identified paralogs, including Atlantic salmon–specific copies, were integrated into the analysis. These were identified using resources such as SalmoBase (http://salmobase.org/) and the NCBI RefSeq databases. Further specifics regarding gene/paralog selection and nomenclature are documented in [31]. The gene accession numbers, symbols, full names, and functional classifications are provided in supplementary file 1. The analysis of mRNA expression levels for these candidate genes involved the application of the NanoString nCounter Analysis technology (NanoString Technologies, Seattle, WA, USA). Probes designed for each gene paralog, aimed at all known transcript variants, were formulated using reference sequences from the NCBI RefSeq database. However, designing salmon paralog-specific probes was not feasible for 58 of the 342 genes due to high sequence similarity between paralogs (see supplementary file 1 for the lists of both paralog-specific and non-paralog-specific probes). Practical execution encompassed the utilization of the nCounter Custom CodeSet for probes and the nCounter Master kit (NanoString Technologies). The RNA from each sample was denatured and hybridized overnight with a total of 342 gene probes (including 9 reference gene probes), using the NanoString nCounter^®^ Gene Expression Assay and analyzed on the nCounter^®^ MAX Analysis System, following the manufacturer’s standard protocol. Subsequent to hybridization, purification and image scanning were performed the following day.

### Data analysis

Among 9 candidate reference genes in the panel, 8 genes, including *actb*, *ef1a* paralogs (*ef1aa*, *ef1ab* and *ef1ac*), *hprt1*, *prabc2* paralogs (*prabc2a* and *prabc2b*) and *rps20*, were selected for data normalization since they showed a low coefficient of variation (CV) values across the samples. The excluded reference gene was *gapdh* as it showed very high variation (CV% > 100), so even though *gapdh* is commonly used as a reference gene in many studies, it seemed to be unsuitable for data normalization in the brain of Atlantic salmon. Following this, the raw count data obtained from NanoString nCounter mRNA expression underwent normalization through RNA content normalization factors, calculated individually for each sample using the geometric mean count values of the selected set of reference genes. After normalization, a quality control assessment was conducted, with all samples successfully passing the predefined threshold using the default criteria of nSolver Analysis Software v4.0 (NanoString Technologies; www.nanostring.com/products/nSolver). During data analysis using the software, the mean of the negative controls was subtracted, and positive control normalization was carried out by utilizing the geometric mean of all positive controls, following the manufacturer’s recommendations. To establish a baseline signal threshold, a normalized count value of 20 was set as the background signal. Consequently, among the analyzed genes, 82 genes displayed an average signal below this threshold across the samples, leading to the consideration of 255 genes for subsequent analyses. Differential expression analysis was executed using the log-linear and negative binomial model (lm.nb function) as integrated within NanoString’s nSolver Advanced Analysis Module (nS/AAM). Inclusion of predictor covariates in the model encompassed the maturation status and genotypes, as guided by nS/AAM’s suggestions. To mitigate multiple hypothesis testing, the Benjamini-Yekutieli method [46] was employed within the software, with an adjusted *p*-value threshold of < 0.05 deemed statistically significant (all expression data and the corresponding statistical analyses are provided in supplementary file 2). In addition, for further exploration, log-transformed expression values were utilized in calculating pairwise Pearson correlation coefficients (r) between the gene expression of each candidate gene and GSI values across the entirety of the samples.

We employed the Weighted Gene Co-expression Network Analysis (WGCNA) R package (version 1.68), using R software version 4.2.1, to construct gene co-expression networks (GCNs), as outlined by Langfelder & Horvath, 2008 [47]. Our primary focus being the comparison of alternative *vgll3* genotypes, we harnessed all samples from both maturation statuses and time-points within each genotype as biological replicates, ensuring robust statistical power for WGCNA. Thus, the analysis was performed using all expressed genes within each genotype, regardless of differential expression between genotypes or time points and log2-transformed normalized NanoString counts were used as input. To ascertain sample relationships, hierarchical clustering of samples based on gene expression was performed. The construction of co-expression networks encompassed the following stages: (1) determination and quantification of gene co-expressions through Pearson correlation coefficients, (2) establishment of an adjacency matrix with a focus on scale-free topology employing the coefficients, (3) computation of the topological overlap distance matrix via the adjacency matrix, (4) hierarchical clustering of genes using the topological overlap distance with the “average” method, (5) identification of co-expressed gene modules through employment of the cutTreeDynamic function, with a minimum module size of 10 genes, (6) allocation of colors to each module and representation of module-specific expression profiles through the principal component (module eigengene), and (7) merging of highly similar modules based on module eigengene (ME) dissimilarity, utilizing a distance threshold of 0.25, to finalize the set of co-expressed gene modules. Furthermore, we implemented a conditional co-expression analysis [48], wherein co-expression networks were individually constructed for each *vgll3* genotype. This approach sought to identify the preservation of *early* maturation genotype (EE) modules within the *late* maturation (LL) network and vice versa. With a softpower of 9, an adjacency matrix was established. Lastly, to assess the preservation of modules’ density and connectivity between the reference dataset (EE) and the query dataset (LL), module preservation statistics were computed using WGCNA, following the methodology outlined by Langfelder, Luo, Oldham, & Horvath, 2011 [49]. A permutation test was implemented to iteratively shuffle genes within the query network, calculating Z-scores. The individual Z-scores from all 200 permutations were summarized into a Z-summary statistic.

To further characterize the co-expression modules identified through WGCNA, we used WebGestalt [50], to assess the genotype-specific similarities and differences in biological processes associated with the genes within each module. The assessment was adjusted for gene-set enrichment analysis at FDR < 0.05, with a specific Gene Ontology/Biological Process (GO/BP) with minimum level 2 threshold for inclusion (level 2 and above: terms of medium to higher specificity in the GO hierarchy). Next, the enriched GO/BPs were compared between the vgll3 genotypes. For the anticipation of potential gene regulatory/molecular connections and the identification of key genes displaying the highest connection count (referred to as regulatory hubs), the differentially expressed genes identified in each comparison were converted to their conserved orthologs in humans using BioMart software [51] and the ENSEMBL gene IDs for Atlantic salmon were used as input (assembly Ssal_v3.1, GCA_021399835.1). These orthologs were chosen due to their extensive validated and studied interactome data across vertebrates. Subsequently, these orthologs were used as input for STRING version 12.0, a comprehensive knowledge-based interactome database for vertebrates [52, 53]. Predicted connections between genes were based on multiple factors such as structural similarities, cellular co-localization, biochemical interactions, and co-regulation. The confidence level for each molecular connection prediction was maintained at a medium setting, which is the default threshold.

## Results

### Differences in gene expression between *vgll3* genotypes

**Fig. 1:**
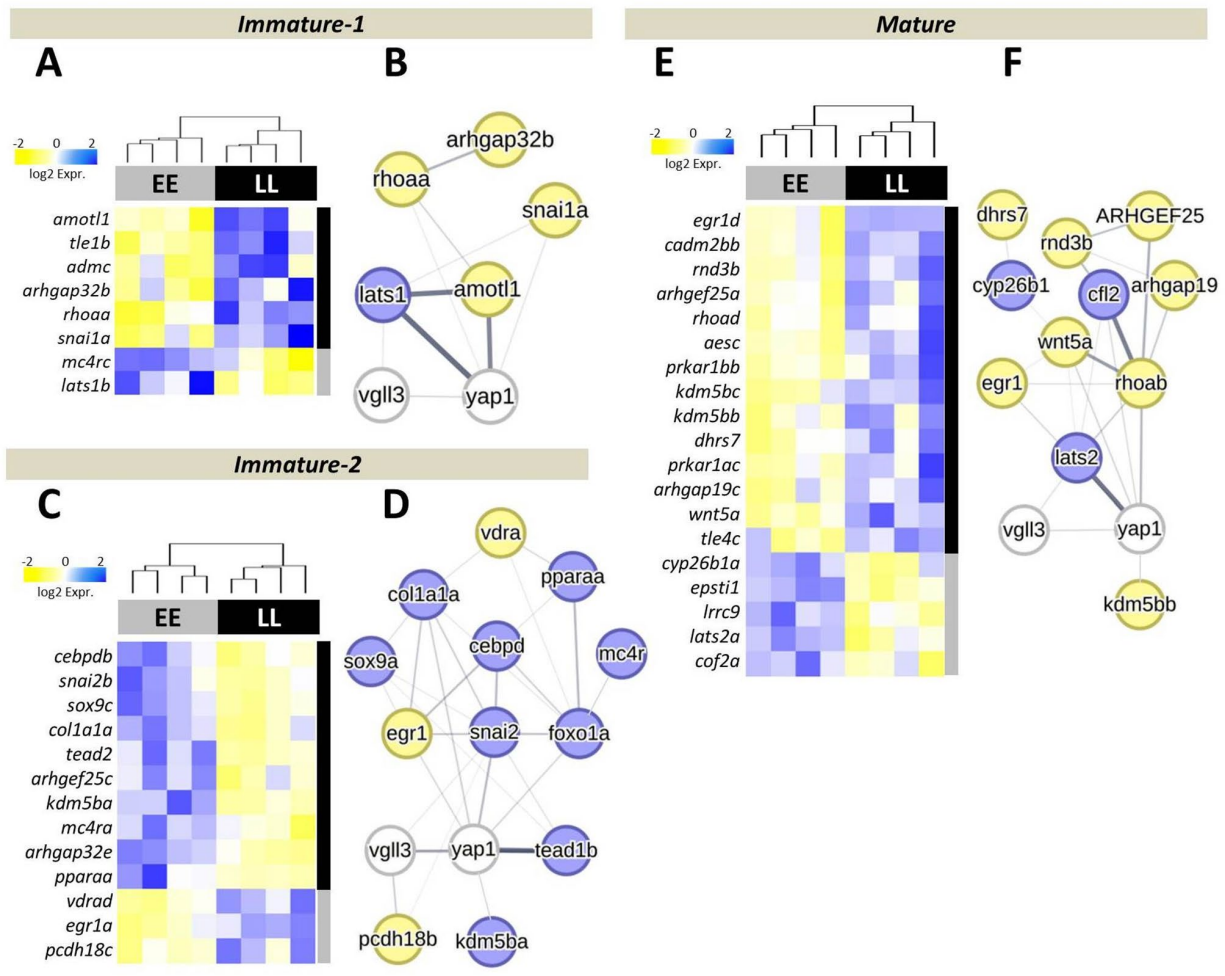
Differential expression of genes between the *vgll3* genotypes and prediction of their regulatory connections in the brain. Heatmaps show differentially expressed genes between *vgll3* genotypes at three timepoints (**A, C, E**) and their potential regulatory connections (**B, D, F**). The width of the lines connecting the genes indicates the likelihood of their interaction. In the predicted network, genes displayed in blue represent higher expression levels, while those in yellow signify lower expression levels in vgll3*EE individuals (corresponding to the colors in panels **A**, **C**, and **E**)

We first evaluated variation in expression between different *vgll3* genotypes during distinct developmental stages: *Immature-1* and *Immature-2* (late spring and summer periods), as well as *Mature* (early autumn) (Fig. 1). We found 8 genes expressed differentially between genotypes at the *Immature-1*, and 13 genes at the *Immature-2* time point, as well as 19 genes at the *Mature* time point. At *Immature-1*, all differentially expressed genes, except *lats1b* and *mc4rc*, exhibited lower expression in vgll3*EE genotype individuals (Fig. 1A). Interestingly, two paralogs of *kdm5b* showed very distinct expression patterns between the genotypes: *kdm5ba* exhibited higher expression in vgll3*EE individuals at the *Immature-2*, whereas *kdm5bb* showed higher expression in vgll3*LL individuals at the *Mature* time-point. The knowledge-based interactome analysis in revealed that while only one gene, *last1b*, had direct molecular/regulatory connection with *vgll3*, four genes showed potential direct molecular/regulatory connection with *yap1* (*amotl1*,*rhoaa*,* lats1b* and *snai1a*) (specified with connecting lines between the genes in Fig. 1B). At *Immature-2*, we found 10 genes with higher expression in vgll3*EE genotype individuals and 3 genes showing higher expression in vgll3*LL genotype individuals (Fig. 1C). The interactome analysis revealed six genes (*col1a1a*, *egr1a*, *foxo1*, *snai2b*, *kdm5ba* and *tead2/tead1b*) with direct molecular/regulatory connection with *yap1* whereas two genes (*snai2a* and *pcdh18*) had direct connection with *vgll3* (Fig. 1D). Furthermore, except for *kdm5ba*, rest of the five genes showing molecular/regulatory connection with *yap1* also formed regulatory hubs with other differentially expressed genes (based on them showing the highest number of connections compared to other genes) (Fig. 1D). Moreover, *erg1a* was the only gene among the six genes showed molecular/regulatory connection with *yap1* showing lower expression in vgll3*EE. Finally, at *Mature*, we found 5 genes with higher expression in vgll3*EE genotype individuals, and 14 genes showed higher expression in vgll3*LL genotype individuals (Fig. 1E). The interactome analysis identified 6 genes (*egr1*, *kdm5bb*, *lats2a*, *rhoa*, *clf2*, and *wnt5a*) with direct molecular/regulatory connection with *yap1*, whereas no gene had direct connection with *vgll3* (Fig. 1F). Except for *lats2a* and *cfl2*, the remaining genes showing direct molecular/regulatory connection with *yap1* had lower expression in vgll3*EE individuals and one of them, *rhoa*, formed a regulatory hub, i.e. had highest number of connections with other differentially expressed genes (Fig. 1F). These findings show that major components of the Hippo pathway, along with several of its interacting factors, are differentially expressed in the brain of early- versus late-maturing male salmon, even before the appearance of visible signs of gonadal maturation.

### Maturation specific gene expression differences

**Fig. 2:**
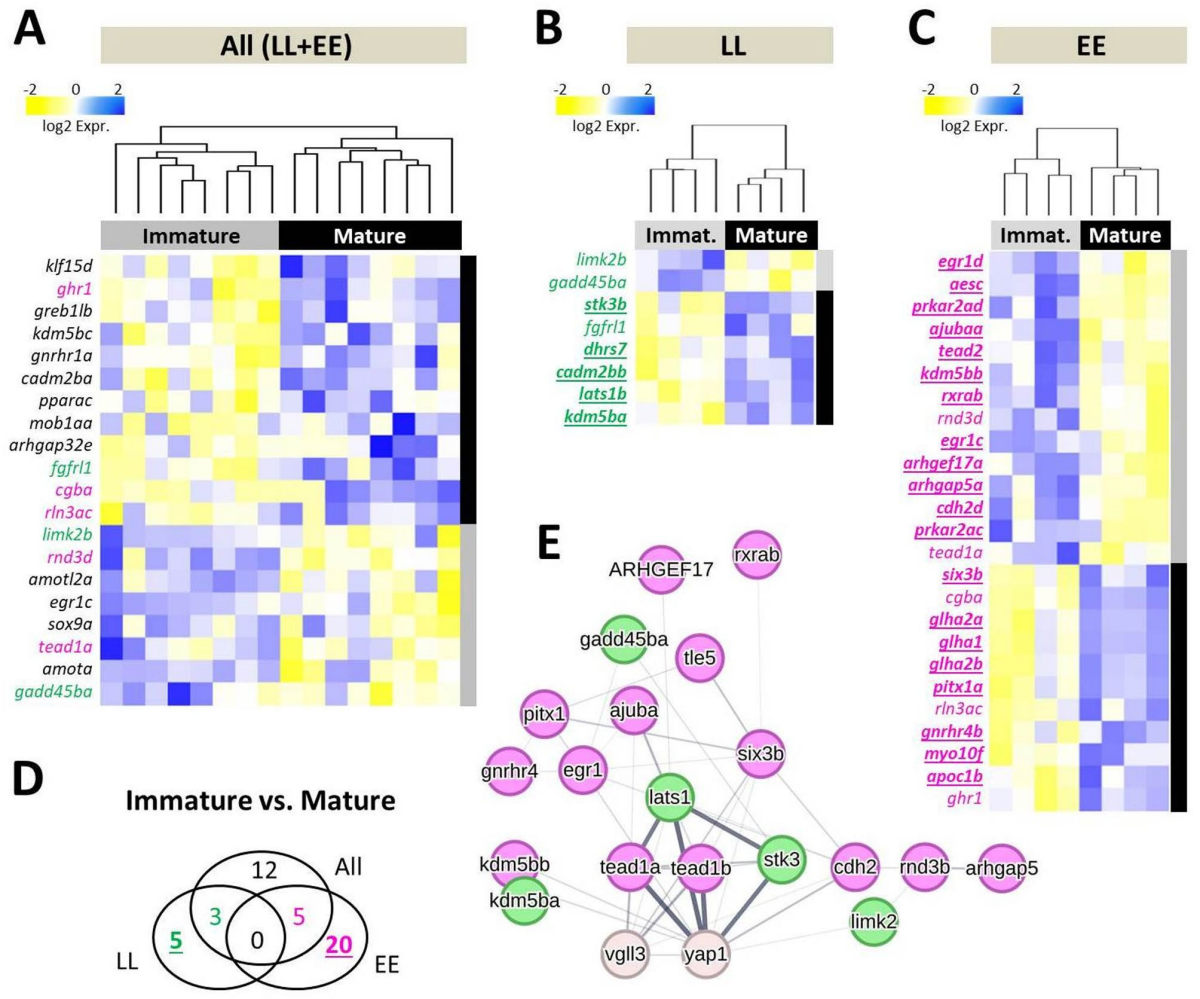
Differential expression of genes between maturity statuses and prediction of their regulatory connections in the brain. Heatmaps showing differentially expressed genes between the maturity statuses in both *vgll3* genotypes (**A**), and within vgll3*LL (**B**) and vgll3*EE genotype (**C**). A Venn diagram representing the numbers of differentially expressed genes which overlap between the comparisons (**D**). The regulatory connections predicted between the genes highlighted in green and magenta represent the overlapping genes shown in the Venn diagram (**E**). The width of the lines connecting the genes indicates the likelihood of their interaction

To pinpoint alterations in gene expression linked to the transition from an immature to a mature state, we compared immature individuals obtained at both *Immature-1* and − *2* time points and those collected at the *Mature* time point. We detected 20 differentially expressed (DE) genes between mature and immature individuals when *vgll3* genotypes were combined (Fig. 2A). The separate comparisons of *vgll3* genotypes revealed 8 DE genes in the vgll3*LL genotype and 25 DE genes in the vgll3*EE genotype between mature and immature individuals, as shown in Figs. 2B and C. In vgll3*LL individuals, 6 out of the 8 and in vgll3*EE individuals 11 out of the 25 DE genes between immature vs. mature individuals had higher expression at the *Mature* time point (Fig. 2B-C). Across the three comparisons of immature versus mature individuals, no gene was found to be differentially expressed in all comparisons (Fig. 2A-D). However, 12 genes were identified as differentially expressed regardless of *vgll3* genotype between immature and mature individuals, as indicated by the black font in Fig. 2A. We proceeded with an investigation of potential functional/molecular connections among differentially expressed genes that exhibited *vgll3* genotype-specific variation in expression (colored numbers in Fig. 2D). The predicted connections between these genes revealed that in the *vgll3* genotype specific comparisons, 5 and 15 DE genes within vgll3*LL and vgll3*EE individuals, respectively, were found to be part of a common molecular/regulatory connection network (colored green and magenta in Fig. 2E). Among the DE genes in vgll3*LL individuals, four genes, *kdm5ba*, *lats1b*, *limk2* and *stk3*, had direct connection with *yap1* and all showed higher expression in the mature stage within this genotype (Fig. 2E). Among the DE genes in vgll3*EE individuals six genes, *cdh2*, *egr1*, *six3b*, *tead1a* and *tead2* (or *tead1b*), had direct connections with *yap1* and also three of them, *six3b*, *tead1* and *tead1b*, had direct connections with *vgll3* (Fig. 2E). Interestingly again, the two paralogs of *kdm5b* showed distinct expression patterns between time points depending on genotype: *kdm5ba* exhibited higher expression in mature individuals with the vgll3*LL genotype, whereas *kdm5bb* showed lower expression in mature individuals with the vgll3*EE genotype. These findings show that more components of the Hippo pathway are differentially expressed in the brain of early-maturing *vgll3* allele carriers across maturation stages.

**Fig. 3:**
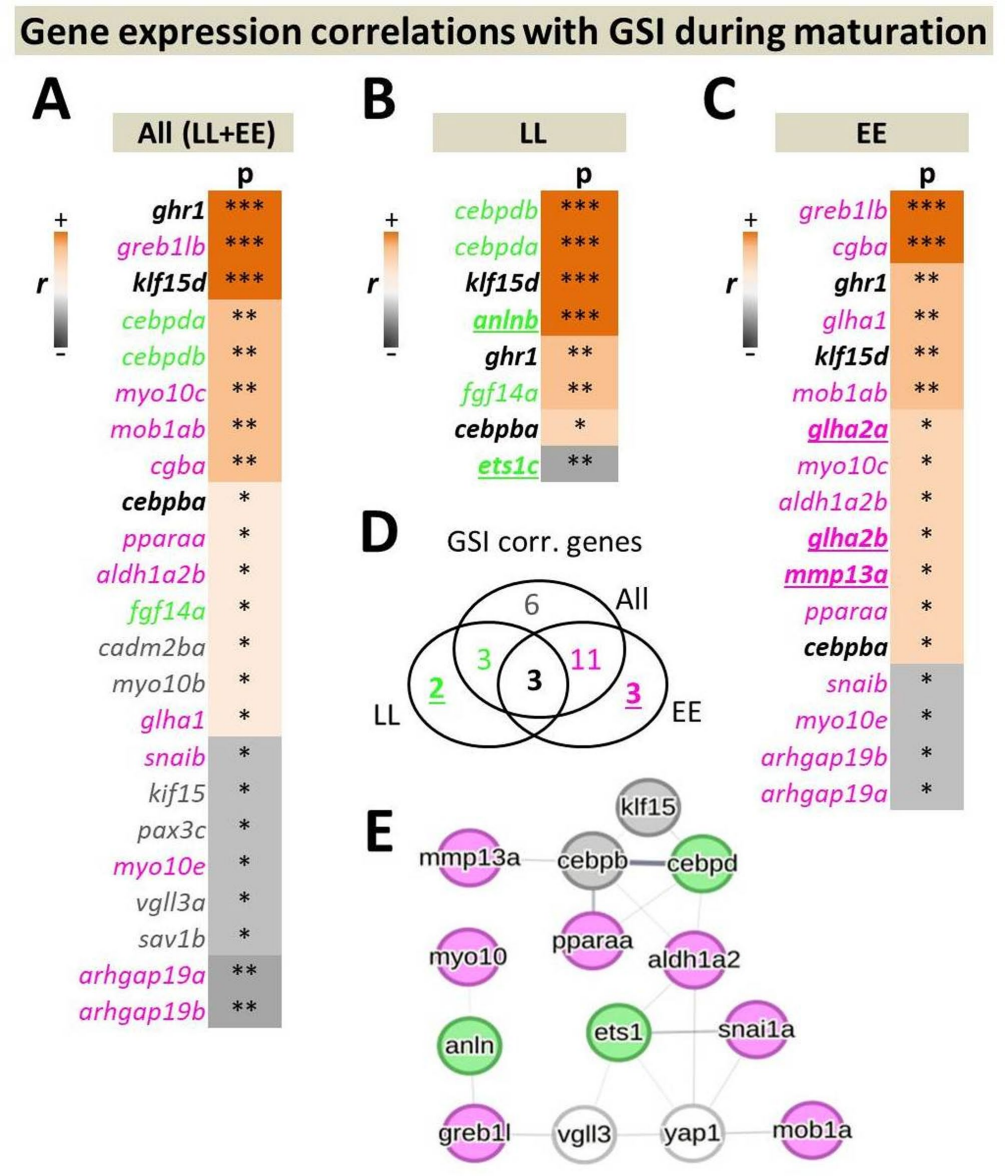
Genes exhibiting expression patterns correlated with GSI and their predicted regulatory connections in the brain. Ranking of significant Pearson correlations between gene expression and GSI in the brains of salmon, encompassing both *vgll3* genotypes (**A**), within vgll3*LL (**B**) and vgll3*EE (**C**) genotype. The Venn diagram illustrates the numbers of genes significantly correlated with GSI that are unique to each comparison or shared between them. (**D**). The regulatory connections predicted between the genes highlighted in green, magenta and grey represent the overlapping genes shown in the Venn diagram (**E**). EE and LL indicate vgll3*EE and vgll3*LL genotypes, respectively, and p and r indicate *p*-values (* < 0.05; ** < 0.01; *** < 0.001) and Pearson correlation coefficient. Gene colors correspond to the color codes within the Venn diagram, and the underscored genes are those that demonstrate expression correlation with GSI in only one of the *vgll3* genotypes. The width of the lines connecting the genes indicates the likelihood of their interaction

Subsequently, we examined the expression of each gene of the panel in correlation with the gonadal development stage (GSI), aiming to uncover genes exhibiting expression profiles within the brain that are closely intertwined directly with the gonadal maturation process. We observed a positive correlation between the expression of three genes—*ghr1*, *klf15d*, and *cebpba*—and GSI, regardless of the *vgll3* genotype. (Fig. 3A-D). Furthermore, two of these genes, *ghr1* and *klf15d*, were among the genes with most significant positive expression correlations within each genotype. Overall, the majority of the correlations detected with GSI were positive, irrespective of the grouping of *vgll3* genotypes (Fig. 3A-C). Four genes amongst vgll3*EE genotype individuals, *arhgap19a/b*, *myo10e* and *snaib*, showed negative expression correlations with GSI, and all were shared between the comparisons of vgll3*EE genotype and both genotypes together. Amongst vgll3*LL individuals, expression of one gene, *ets1c*, was found to be negatively correlated with GSI (Fig. 3B). The predicted molecular/regulatory connections between the genes showing genotype-specific expression correlations (colored numbers in Fig. 3D) revealed a potential network between three and seven genes, respectively, for vgll3*LL and vgll3*EE genotype individuals, as well as two overlapping genes across all groups (Fig. 3E). For vgll3*EE genotype individuals, three genes (*aldh1a2*, *snai1a* and *mob1ab*) showed direct connections with *yap1* and one gene (*greb1lb*) with *vgll3*, whereas for vgll3*LL genotype one gene, *ets1*, showed direct connection with both *vgll3* and *yap1*. These findings further show that more interacting partners of the Hippo pathway exhibit expression changes that correlate with gonadal maturation in the brain of early-maturing *vgll3* allele carriers.

### Identification of gene co-expression networks

To attain a more comprehensive understanding of the transcriptional dynamics involving components of the Hippo pathway and their established interacting genes, we employed network-based co-expression analyses. This approach facilitated the monitoring of genotype-specific alterations within each network. To execute this strategy, we initially constructed gene co-expression networks (GCN) in the brain of each *vgll3* genotype. Subsequently, we evaluated the extent to which the identified gene co-expression modules were conserved between the genotypes. In essence, we established the GCN within one genotype (vgll3*EE or vgll3*LL) and subsequently tested the preservation of its modules within the other genotype (vgll3*LL or vgll3*EE), respectively.

**Fig. 4:**
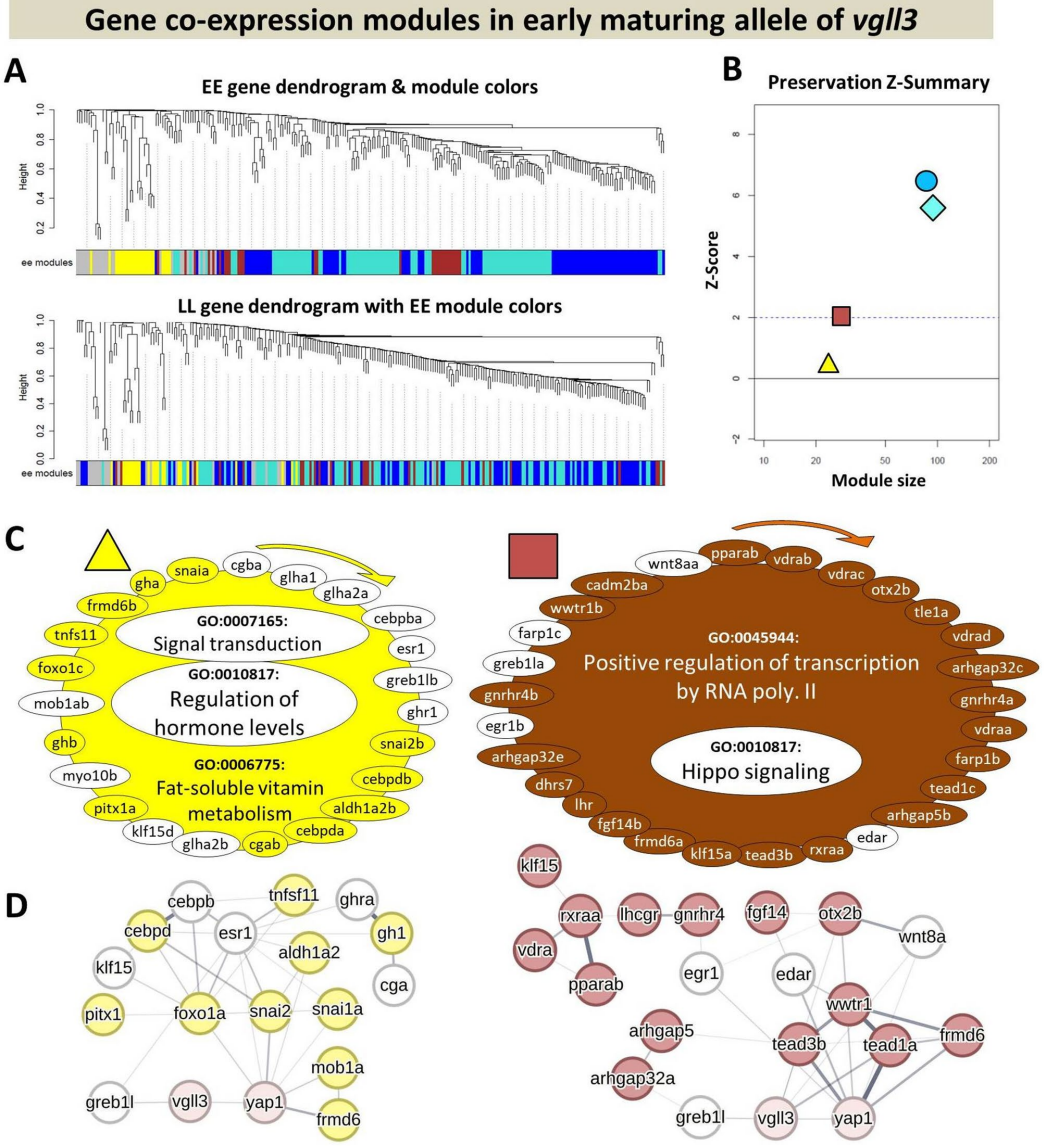
Depiction of co-expression analysis of the vgll3*EE genotype in the brain. (**A**) The visual representation shows vgll3*EE module preservation in vgll3LL individuals. Dendrograms illustrate an average linkage clustering tree based on topological overlap in gene expression profiles, thus showing that components of certain modules in one genotype (EE) are scattered and unpreserved in the other (LL), indicating low preservation across groups. The colors in the lower section of the dendrograms correspond to vgll3*EE clustered co-expression modules. The top section displays vgll3*EE modules with their respective colors, while the bottom section represents the strength of vgll3*EE module gene preservation in vgll3*LL genotype individuals. (**B**) Preservation Z-summary scores in the vgll3*LL for vgll3*EE modules (colors represent vgll3*EE modules). Z-summary < 2 represents the absence of preservation (dotted blue line) and Z-summary between 2 and 10 implies moderate preservation. (**C**) The genes within each of the two identified vgll3*EE genotype modules exhibit the lowest preservation in the vgll3*LL genotype. Genes within each module that are uncolored lack preserved expression correlations in vgll3*LL individuals. The clockwise arrows above each module denote the sequence from highest to lowest expression correlations among genes within that module. In each module, the GOs that appear uncolored lost their enrichment status once the genes without color were excluded. (**D**) Predicted regulatory interactions among the genes within each of the modules showing lower preservation. The width of the lines connecting the genes indicates the likelihood of their interaction

We identified 4 modules within vgll3*EE GCN (Fig. 4A and B) of which 2 modules, brown/square and yellow/triangle, showed low preservation (Z-summary < 2) in vgll3*LL, i.e. some of the genes in each module do not have significant expression correlations in vgll3*LL genotype (Fig. 4A). For each module, we performed an enrichment analysis of Gene Ontology (GO) Biological Processes to identify the major processes in which the genes within each module are engaged. In the yellow/triangle module, we found associations with processes of regulation of hormone levels, signal transduction and fat-soluble vitamin metabolism (Fig. 4C). The yellow/triangle module showed the least preservation between the genotypes with 11 out of its 23 genes showing no co-expression preservation in vgll3*LL genotype individuals (genes with white background in each module in Fig. 4C). In the brown/square module, we found associations with processes of positive regulation of transcription and the Hippo pathway signaling and only 5 out of 25 genes showing no co-expression preservation in vgll3*LL genotype individuals (Fig. 4C). The removal of non-overlapping genes in the yellow/triangle and brown/square modules led to loss of significance of two GOs in yellow/triangle and one GO in the brown/square module; regulation of hormone levels and signal transduction in the yellow/triangle and the Hippo signaling in the brown/square modules (non-colored GOs in Fig. 4C).

We queried the STRING interaction database with module genes to identify potential molecular/regulatory connections among them, along with the identification of hub genes exhibiting the greatest number of interactions. The prediction of molecular/regulatory connections between the genes within each low preserved module revealed that in both modules at least one gene among those not showing co-expression preservation had direct connection with *vgll3* (represented with lines directly connecting the non-colored genes with *vgll3*) (Fig. 4D). Interestingly, in each module a different paralog of *greb1l* (*greb1lb* in the yellow/triangle and *greb1la* in the brown/square modules) was in direct interaction with *vgll3* but lacking co-expression preservation in vgll3*LL genotype individuals (Fig. 4D). Moreover, in each module one or two genes with direct connection with *yap1* also lacked co-expression preservation in vgll3*LL genotype individuals (*esr1* in the yellow/triangle and *egr1* and *edar* in the brown/square modules) (Fig. 4D).

**Fig. 5:**
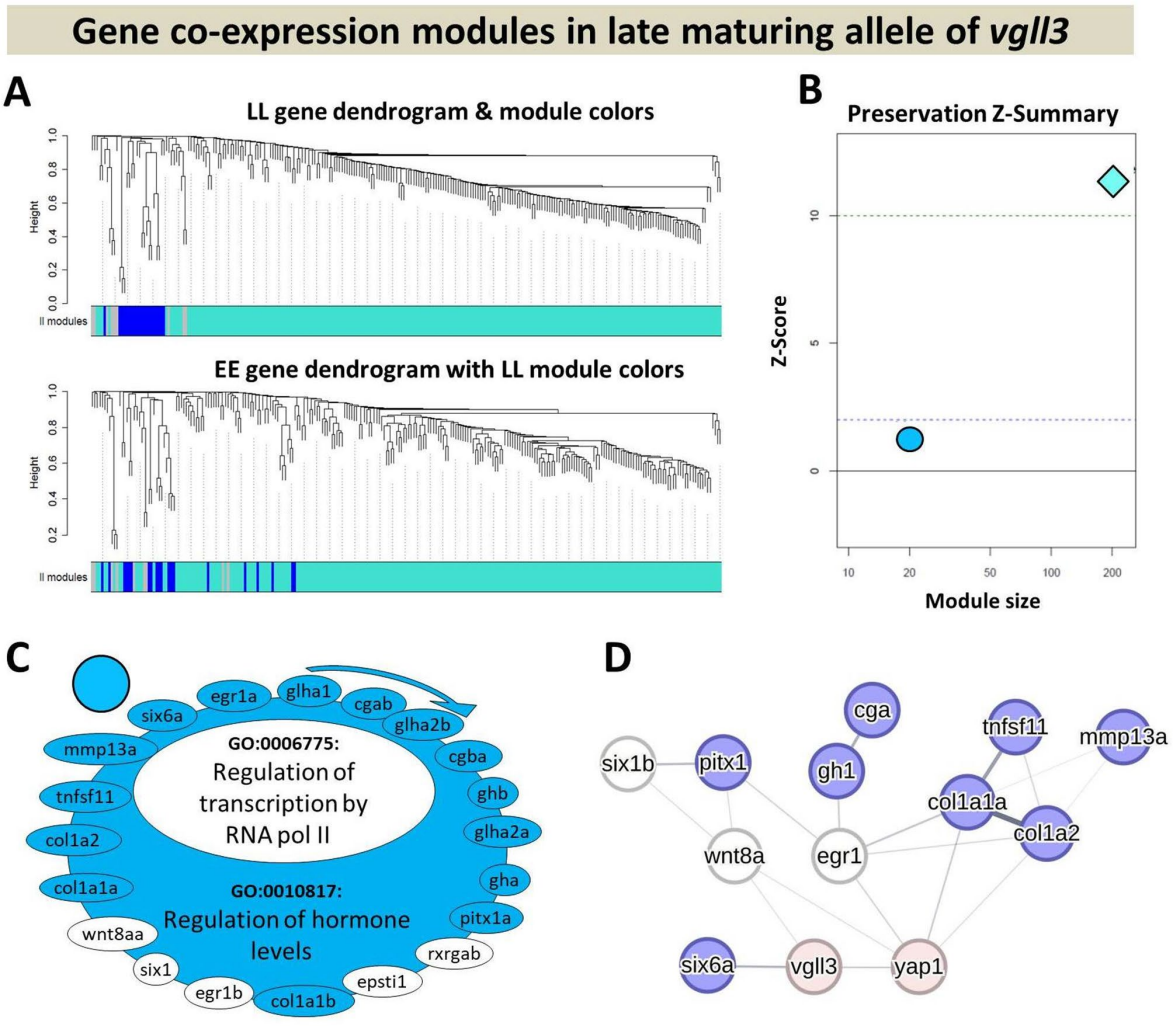
Depiction of co-expression analysis of the vgll3*LL genotype in the brain. (**A**) Dendrograms display clustering and preservation of vgll3*LL modules in vgll3*EE individuals highlighting that components of certain modules in one genotype (LL) are scattered and unpreserved in the other (EE), indicating low preservation across groups. (**B**) Z-summary scores indicate lack of preservation (< 2) and moderate preservation (2 to 10) for vgll3*LL modules in vgll3*EE. (**C**) The genes in the least preserved vgll3*LL module in vgll3*EE. Non-preserved genes are shown without color, and clockwise arrows indicate directions of gene expression correlation. Enriched GO terms are listed, highlighting those no longer enriched after removing non-colored genes. (**D**) Predicted interactions within the less preserved (blue/circle) module. Line thickness is relative to interaction probabilities. The technical/methodological details of each panel follow those described in Fig. 4

In vgll3*LL individuals, we found only 2 modules and one of them (the blue/circle module) showed a low level of preservation (Z-summary < 2) compared to vgll3*EE individuals (Fig. 5A and B). The other module (turquoise/rhombus module) was very large with 215 co-expressed genes and also with the highest preservation level between the genotypes (Z-summary > 10). The blue/circle module included genes involved in positive regulation of transcription and regulation of hormone levels (Fig. 5C), indicating a potential regulatory connection between genes within these processes in vgll3*LL individuals. We observed that the regulatory connection in the vgll3*EE genotype was no longer present after some of the genes in the blue/circle module showed no expression correlation with the rest of the genes within this module (genes with white background in Fig. 5C). Conversely, the increase in transcriptional activity in vgll3*EE appears to be linked to genes encoding components of the Hippo pathway (Figs. 4C and 5C). In other words, the activity of the Hippo pathway may be less impacted and/or may play a less extensive role in the brains of vgll3*LL individuals compared to vgll3*EE individuals during sexual maturation. The prediction of molecular connections between the genes within the blue/circle module revealed that two genes, *six6a* and *wnt8a*, have direct connections with *vgll3* and four genes, *col1a2*, *col1a1a*, *egr1* and *wnt8a*, have direct connections with *yap1*. However, only two of these genes, *egr1 and wnt8a*, have lost their co-expression status in vgll3*EE (Fig. 5D).

## Discussion

We investigated how different genotypes of *vgll3* gene, a key determinant of sexual maturity age in Atlantic salmon [16], as well as a critical co-factor of the Hippo pathway [37], correlate with transcriptional activity in the brain prior to and after sexual maturation. We explored whether these transcriptional changes in the brain could reflect a potential role for the Hippo pathway in early maturation before any visible signs of maturation emerge in the testis. This is particularly interesting because the molecular signal regulated by *vgll3* is active and plays different roles in all the brain regions involved in sexual maturation [54–56]. In addition, the Hippo pathway is a mediator of fatty diet-induced transcriptional changes in different tissues including brain and particularly hypothalamus [39]. We studied the expression variation of genes encoding components of the Hippo pathway and their associated interacting partners/signals, along with genes involved in sexual maturation, in the brains of individuals with different *vgll3* maturation alleles (*early* or *late*) using the NanoString platform. This enabled us to identify components of the Hippo signaling pathway that may contribute to central nervous system regulation of maturation in Atlantic salmon, a species with a breeding strategy closely linked to seasonal changes and adiposity.

### *vgll3*-dependent differential expression of Hippo pathway components in the brain prior to the onset of puberty

We found *lats1b*, a paralog of a gene encoding a major kinase in the Hippo pathway to have higher expression in the brain of individuals with the vgll3*EE genotype in the earliest immature stage samples (*Immature-1*). At the core of the Hippo pathway lies a series of kinase reactions [31]. Activation of major kinases (LATS1/2) in the Hippo pathway phosphorylates and deactivates YAP1 and thus prevents the movement of YAP1 into the nucleus, thereby regulating the expression of critical genes associated with cell division, apoptosis, and cell migration [57]. Although this molecular process is highly conserved in animals [57], it is not known if the suppression of YAP1 activity by LATS1 plays a role in early signals of triggering sexual maturation in the brain. In rat, high fat diet decreases the level of YAP1 activity and subsequently increases the neural differentiation in the hypothalamus [39]. Our results indicate that individuals with the vgll3*EE genotype may have an overall higher level of neural differentiation due to lats1b mediated inactivation of yap1 in the brain. However, further cellular and histological investigations are required to validate whether there is a potential difference in neural differentiation between the genotypes in brain regions involved in the central control of maturation, such as the hypothalamus. This is particularly important because hypothalamic neural differentiation is an essential cellular process prior to puberty, which leads to fully active HPG axis and the downstream neuroendocrine cascade ensuring gonadal activation [58].

Another relevant finding was higher expression of *mc4rc*, a paralog of the gene encoding a membrane-bound receptor and member of the melanocortin receptor family, in the brain of individuals with vgll3*EE genotype again at *Immature-1* (similar to *lats1b*). In certain fish species, such as *Xiphophorus* swordtails and medaka, the Melanocortin 4 receptor (*Mc4r*) has been linked to the initiation of puberty [59, 60]. Mc4r belongs to the class A of G-protein coupled receptors (GPCR) and plays a role in maintaining energy balance. Notably, in humans, mutations in the *MC4R* gene represent the most prevalent monogenic cause of severe early-onset obesity [61]. Our results suggest that increased expression of *mc4rc* in the brain of immature males might be a sign for early maturation long before changes in gonads, however, the potential molecular link between *vgll3-*mediated activation of the Hippo pathway and *mc4r* has not been investigated in any species. Finally, we also found reduced expression of two major factors, *snai1* and *rhoaa*, in vgll3*EE genotype individuals. Snai1 is an activator of Yap1, and thus may lead to similar results as above, i.e., early onset of maturation, but independent of lats1 [62]. The function and expression of *rhoaa* is suggested to be inhibited by *vgll3*-dependent activation of the Hippo pathway [31]. Mutations in *RHOA* have been recently suggested to be linked with central precocious puberty in humans [63], suggesting that its increased brain expression may be a conserved molecular indicator of early maturation in the vertebrate central nervous system.

### Distinct interacting partners of the Hippo pathway may participate in the onset of puberty in the brain

In the later immature time-point, *Immature-2*, close to the onset of puberty (from no signs to initial signs of gonadal changes), we found a major component and several interacting partners of the Hippo pathway to be differentially expressed. A major transcription factor of this pathway, *tead2*, had higher expression in the brain of individuals with vgll3*EE genotype. *Tead2* is one of the highly expressed components of the Hippo pathway in mouse brain and its expression is associated with neural cell proliferation [64]. One of the interesting interacting partners of the Hippo pathway with higher expression in the brain of vgll3*EE individuals was a gene encoding a paralog of Forkhead box protein O1 (*foxo1c*). In mouse, *FOXO1* encodes a transcription factor that participates in regulation of the onset of puberty by affecting the release of GnRH in the hypothalamus [65], however, such a role has, to our knowledge, not yet been reported in fish species. Two other interacting partners of the Hippo pathway with potential roles in central regulation of the onset of puberty were *kdm5b (jarid1b)*, which has been shown to be modified epigenetically prior to puberty in the rat brain [66], and *mc4ra*, another paralog of Melanocortin 4 receptor (*MC4R*) with conserved role in stimulating the central initiation of the puberty (see above). Yet again the findings in mammals about these two genes remained to be investigated in teleost fish. Importantly, we also found two paralogs of the *kdm5b* gene (*kdm5ba* and *kdm5bb*) to exhibit distinct, genotype-specific expression patterns between immature and mature individuals, suggesting potential paralog-specific sub- or neo-functionalization in Atlantic salmon that should be explored further.

The only interacting partner of the Hippo with lower expression in vgll3*EE individuals was a paralog encoding an immediate early gene (IEG) called early growth factor-1 (*egr1a*). Strikingly, *egr1* is known to be a major factor mediating socially-induced sexual maturation in fish hypothalamus [67]. We have recently found *egr1* differential expression as part of a pituitary gene network associated with *vgll3*-dependent transcriptional regulation of gonadotropins [29]. However, since *egr1* is a positive regulator of sexual maturation in the HPG axis, its higher expression in vgll3*LL individuals may indicate a different path for individuals with *late* alleles in sexual maturation which may more relate to social interactions. A recent study demonstrated that Atlantic salmon with the vgll3*LL genotype show higher levels of aggression [68], and interestingly, the socially induced sexual maturation through *egr1* is linked to social interactions involving aggressive/submissive (rather than cooperative) behaviors in cichlid fish [69]. Taken together, these findings may suggest different interacting partners of the Hippo pathway participating in the onset of *vgll3*-dependent sexual maturation in Atlantic salmon.

### Maturation progression involves different components of the Hippo pathway in alternative *vgll3* genotypes

Our expression comparison between immature and mature stages suggests that distinct components of the Hippo pathway are involved in the maturation process in the alternative *vgll3* homozygotes. We found that in vgll3*LL individuals during the transition, two genes encoding major components of the Hippo pathway, *lats1b* and *stk3b*, are predicted to have direct molecular/regulatory connections with *yap1* and also showed higher expression in the mature than the immature stage. LATS1 is a critical regulator of YAP1 activity as described above [57]. While its expression was higher in vgll3*EE individuals at the immature stage, its role seems to be important during maturation in vgll3*LL individuals indicating a time-dependent expression shift (heterochrony) for *lats1b* in the brain of individuals with distinct genotypes, and suggesting a potential heterochrony in the neural differentiation [39]. Interestingly, STK3 (*stk3a* in salmon) encodes a highly conserved activator of LATS1 (so called MST2), which is essential for LATS1 mediated inhibition of YAP1 [70], and therefore may provide an evidence for presence of mst2-last1b-yap1 regulatory axis in the brain of vgll3*LL individuals upon entrance to maturation. However, the presence of such a regulatory axis in the brain needs to be further investigated with functional approaches. During the transition to maturation in the brain of vgll3*EE individuals, two other major regulators of the Hippo pathway, *tead1* and *tead2*, which also have direct molecular/regulatory connection with both *vgll3* and *yap1*, had reduced expression suggesting lower level of neural cell proliferation (but not differentiation) in the mature stage [64]. However, it should be noted that the roles of *tead1* and *tead2* in neural cell proliferation and differentiation have not been investigated at the cellular level in the brain of any fish species and have only been demonstrated in mammalian models [64].

### Study limitations and future directions

#### Technical limitations

NanoString technology was initially developed for applications such as studying rare tumors, where sample availability is extremely limited, making its attributes particularly valuable [71]. Its high sensitivity and precision, combined with the ability to directly measure RNA levels without amplification, minimize the risk of false positives and false negatives. These features make NanoString a good method for analyzing gene expression in precious samples with scarce biological replicates, allowing eco-evo researchers to obtain reliable and accurate data even in challenging scenarios. However, there are several limitations to this technique that should be noted.

- This approach is most suitable when the gene(s) underlying the phenotype of interest and their functions are well-characterized, as is the case in our study with *vgll3*, a known co-factor of the Hippo pathway. It is particularly useful when the goal is to explore the dynamics of a specific, related pathway in high resolution.
- In Atlantic salmon, which has undergone an additional whole-genome duplication (WGD), the presence of multiple highly similar paralogs presents challenges for probe design. In some cases, even the 3’-UTRs of paralogs are nearly identical, making it difficult to achieve paralog-specific targeting [72].
- In our study, we included genetic markers as positive controls on the NanoString platform. For future applications, it would be valuable to identify and incorporate genes that are universally expressed across different regions of the salmon brain to further strengthen positive control design.

#### Functional validity and data interpretations

Our study relies solely on gene expression analysis of the Hippo pathway. Given the pathway’s complexity, particularly at the level of protein-protein interactions, it is important that future studies validate these findings at the protein level. Moreover, as we used whole brain tissue for our assessments, the observed expression changes should be further validated at a brain region-specific level and complemented with cellular and histological analyses.

It is also important to note that although the three time points included in this study were selected based on our previous findings at these stages [14, 21, 25, 26], future studies would benefit from inclusion of additional time points. This study builds upon our earlier work in which distinct *vgll3* genotype-dependent differences in adipose tissue gene expression and lipid profiles were observed, supporting the relevance of these selected stages for further investigation [14, 21, 25, 26]. However, to fully understand the dynamic regulation of the Hippo pathway before and during maturation, inclusion of additional time points would be beneficial, particularly for capturing transient transcriptional changes in pathway components.

Lastly, the predicted molecular interactions presented in this study are based on knowledge-driven data from the zebrafish model, which possesses fewer paralogs than salmon due to the absence of the additional WGD event seen in salmonids [72]. This limits the interpretation of our findings, especially regarding potential paralog-specific sub- or neo-functionalization [73, 74]. These limitations highlight the need for developing knowledge-based interactome databases specifically tailored to teleosts, particularly salmonids with an additional WGD event [53].

## Conclusions

This study highlights the potential ecological and evolutionary significance of brain gene expression patterns in shaping genotype-linked life history strategies, such as sexual maturation, in Atlantic salmon. It reveals that early maturation might be reflected at the central nervous system level, with distinct transcriptional signatures in the Hippo pathway preceding visible gonadal changes. These transcriptional differences among individuals with *early*- and *late*-maturing *vgll3* genotypes suggest that the Hippo pathway may participate in the evolutionary adaptation of maturation timing. Interestingly, while expression differences in Hippo components persist across maturation stages, the specific genes involved vary within and between genotypes, reflecting a dynamic interplay between these genetic factors that may influence maturation timing. These findings propose a potential role for the Hippo pathway in coordinating genotype-driven variation in sexual maturation at the brain level in Atlantic salmon. However, further functional studies are needed to validate these molecular connections and their broader implications for life history evolution.

## Electronic supplementary material

Below is the link to the electronic supplementary material.

**Supplementary Material 1**: **Supplementary File 1.** Information about the gene probes on the NanoString panel.

**Supplementary Material 2**: **Supplementary, File. 2:** Expression data and statistical analysis.

## Data Availability

Data is provided within the manuscript or supplementary information files.
